# Long term substrate reduction therapy with ezetimibe alone or associated with statins in three adult patients with lysosomal acid lipase deficiency

**DOI:** 10.1186/s13023-018-0768-8

**Published:** 2018-01-27

**Authors:** Maja Di Rocco, Livia Pisciotta, Annalisa Madeo, Marta Bertamino, Stefano Bertolini

**Affiliations:** 1 Department of Pediatrics, Unit of Rare Diseases, Giannina Gaslini Institute, Largo Gaslini 3, 16147 Genoa, Italy; 2 Department of Internal Medicine, University of Genoa, Viale Benedetto XV 6, 16132 Genoa, Italy

**Keywords:** Lysosomal acid lipase deficiency, Ezetimibe, Substrate reduction therapy

## Abstract

**Background:**

Lysosomal acid lipase deficiency is an autosomal recessive metabolic disease with a wide range of severity from Wolman Disease to Cholesterol Ester Storage Disease. Recently enzyme replacement therapy with sebelipase alpha has been approved by drug agencies for treatment of this lysosomal disease.

Ezetimibe is an azetidine derivative which blocks Niemann Pick C1-Like 1 Protein; as its consequence, plasmatic concentration of low density lipoproteins and other apoB-containing lipoproteins, that are the substrate of lysosomal acid lipase, are decreased. Furthermore, ezetimibe acts by blocking inflammasome activation which is the cause of liver fibrosis in steatohepatitis and in lysosomal storage diseases.

**Results:**

Two patients with Cholesterol Ester Storage Disease were treated with ezetimibe for 9 years and a third patients for 10 years. Treatment was supplemented with low dose of atorvastatin in the first two patients during the last 6 years. All patients showed a significant reduction of alanine aminotransferase, cholesterol and triglyceride. Furthermore, no progression of liver fibrosis was demonstrated.

**Conclusion:**

In this observational case series, ezetimibe is effective, safe, and sustainable treatment for lysosomal acid lipase deficiency. Further studies are warranted to demonstrate that ezetimibe is an alternative therapy to enzyme replacement therapy.

## Background

Lysosomal Acid Lipase Deficiency (LAL-D) is an autosomal recessive disease caused by pathogenic variants of the *LIPA* gene leading to absent or decreased activity of LAL enzyme, which results in a progressive lysosomal accumulation of cholesteryl esters (CE) in hepatocytes, adrenal glands, intestines and macrophage-monocyte cells [1].

Diseases secondary to LAL-D are a continuum, classically divided into the complete LAL deficiency, causing infantile-onset LAL-D (formerly known as Wolman Disease, WD), and the LAL deficiency with residual enzymatic activity leading to a childhood/adult-onset form (formerly known as Cholesteryl Ester Storage Disease, CESD).

WD has its onset in the first month of life and it is characterized by a progressive course leading to death before 1 year of age [1]. Hepatosplenomegaly, steatorrhea, abdominal distension, severe malabsorption with resultant malnutrition, and adrenal insufficiency with adrenal calcification are the cardinal features of WD [2].

CESD has a less severe clinical course; isolated hepatomegaly or hepatosplenomegaly, increased plasma transaminases, type IIa or IIb hypercholesterolemia, and hypoalphalipoproteinemia are the more common initial presentations. Hepatic fibrosis with unpredictable progression to micronodular cirrhosis, as well as premature atherosclerosis, can occur [3]. Intermediate clinical presentations are also reported, as well as prenatal forms characterized by non-immune fetal hydrops [1] and asymptomatic elderly forms. [4].

In LAL-D, the typical plasma lipid profile, characterized by increased levels of total cholesterol (TC), low density lipoprotein cholesterol (LDL-C) and triglycerides (TG), and decreased levels of high density lipoprotein cholesterol (HDL-C), is due to complex secondary metabolic alterations.

LAL cleaves CE and TG of apoB-containing lipoproteins (chylomicron remnants, intermediate density lipoproteins and low density lipoproteins), which are recognized by a receptor localized on the cell membrane, internalized by endocytosis, fused to endosome and carried to lysosome; after enzymatic degradation, free cholesterol and fatty acids are released by lysosome [5].

In LAL-D, the reduced efflux of free cholesterol from lysosome causes up-regulation of hydroxymethylglutaryl-CoA reductase in the liver with increased cholesterol synthesis and production of Very Low-Density Lipoprotein (VLDL), as well as dysregulation of Low-Density Lipoprotein Receptor (LDLR) expression, possibly leading to reduced cholesterol clearance [5, 6]. Furthermore, decreased expression of the adenosine triphosphate-binding cassette transporter A1(ABCA1), and consequently reduced efflux of cholesterol to lipid-free or lipid-poor apolipoprotein A-I (nascent HDL), is the cause of reduced levels of HDL-C. [7].

The only therapeutic option had been liver transplantation for many years, being statin therapy unable to prevent liver fibrosis progression toward liver failure. Recently, the enzyme replacement therapy (ERT) with sebelipase alpha has been approved by Food and Drug Administration and European Medical Agency as safe and effective treatment for LAL-D [8]. However, important unanswered questions remain, such as if a long term treatment with sebelipase alfa could modify hepatic consequences of LAL-D, including liver fibrosis, cirrhosis or need of liver transplant.

Ezetimibe, an azetidine derivative, blocks Niemann Pick C1-like 1(NPC1L1) protein which plays a critical role in intestinal cholesterol absorption and in enterohepatic circulation of cholesterol. Consequently, it decreases plasmatic concentration of Low Density Lipoprotein and other apoB-containing lipoproteins, that are the substrate of lysosomal acid lipase [9]*.*

Here we report our experience on long-term treatment with ezetimibe alone or in association with statin in three patients with LAL-D, and we suggest the role of this drug as substrate-reduction therapy.

## Methods

This study is an observational case series and therefore no controls are included. The patients consented publication for their clinical and genetic data.

Demographic, genetic and biochemical data of patients at the time of diagnosis are reported in Table 1.

**Table 1:** Demographic, clinical, biochemical and genetic data at the diagnosis

	Sex	Age at symptoms onset (years)	Signs and symptoms	Age at diagnosis (years)	*LIPA* pathogenic variants	Enzyme activity	ALT (UI/L)	Cholesterol (mmol/L)	TG (mmol/L)
1	F	7.5	Severe hepato-splenomegaly	9.2	c.894G > A //c.652 C > T	2,5^a^	134	7.26	1.36
2	F	9.9	Mild hepato-splenomegaly	9.9	c.894G > A //c.652 C > T	1,6^a^	137	7.83	2.28
3	M	18	Mild hepato-splenomegaly	20.5	c.894G > A//c.894G > A	0.03^b^	115	7.70	1.20

^a^LAL activity in blood lymphocytes (Reference values: 56.1 ± 7.6 nmol/mg protein/h);^b^ LAL activity in Dried Blood Spot; (Reference values: 0.8–3.0 nmol/punch/h); ALT reference 10–45 U/L, Cholesterol reference value below 5.17 mmol/L, Triglycerides reference value 0.3–1.7

**Fig. 1:**
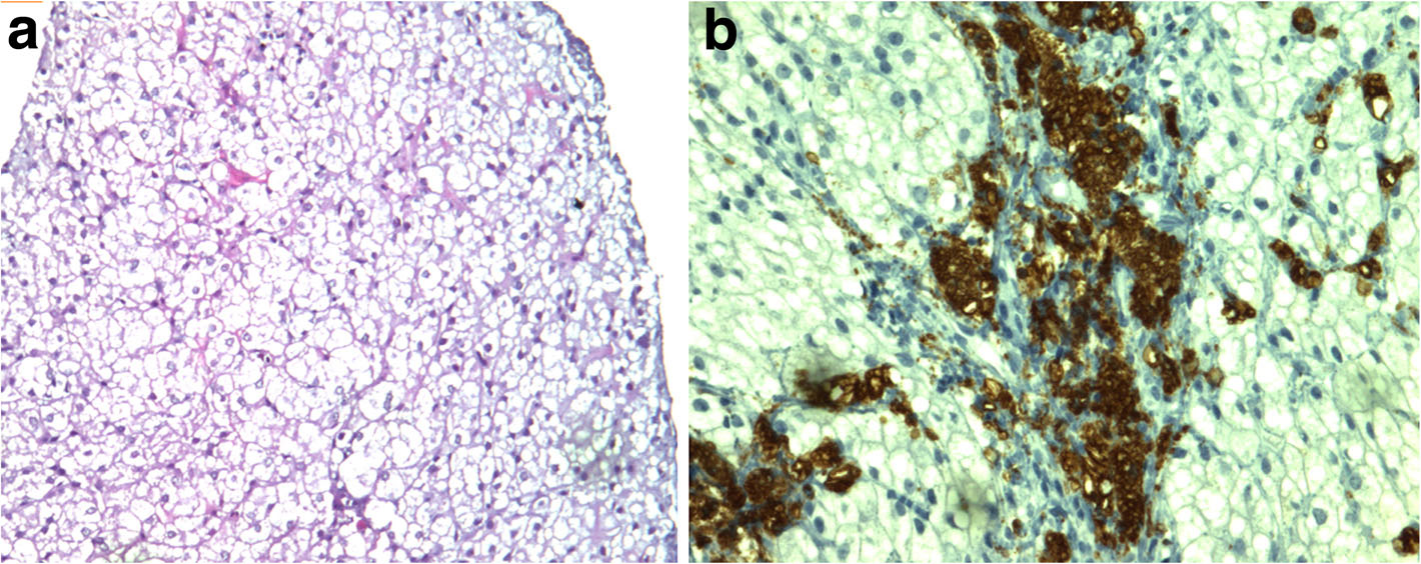
**a** Liver histopathology (H&E 10 X). H&E shows uniform microvesicular steatosis in hepatocytes. **b:** Immuno histochemical stain for CD68 shows portal infiltration of macrophages with lipid storage

Patient 1 was born from unrelated parents; no family history of dyslipidemia was reported. She was diagnosed at the age of 9, when she was admitted to the hospital presenting with hypercholesterolemia, severe hepatosplenomegaly, and hyperechoic liver on ultrasound, that were detected during previous investigations for autoimmune thrombocytopenia and were persistent after normalization of platelets count. Light-microscopy and immunohistochemistry of liver biopsy showed massive vesicular steatosis, portal and septal fibrosis, portal infiltration of macrophages (CD68-positive), and lymphoid cells with abnormal lipid deposits and lipid droplets in hepatocytes and Kupffer cells (Fig. 1). Electron microscopy demonstrated lysosomal lipid storage in hepatocytes, macrophages, and Kupffer cells, as well as the presence of birefringent cholesteryl ester crystals in hepatocytes. She was initially treated with low-fat diet and food supplements aiming to lowering cholesterol levels. Subsequently, at the age of 18, she started ezetimibe at daily-dose of 10 mg. After 3 years, ezetimibe was associated with low dose of atorvastatin (10 mg/day) to obtain a further reduction of plasma lipids level. No adverse events were recorded during lipid-lowering treatment. No other drugs were used. The score of Alcohol Use Disorders Identification Test (AUDIT) questionnaire at the age of 22 was 3 at the age of 28 was 2 (a score of 7 or more in women indicates a strong likelihood of hazardous or harmful alcohol consumption).

As her dizygotic twin sister was diagnosed with LAL-D, patient 2 was examined as well. She was clinically asymptomatic, but mild hepatosplenomegaly associated with mixed hyperlipidemia was present and enzyme assay and molecular analysis confirmed the diagnosis of LAL-D. As her twin sister, she was initially treated with low-fat diet and food supplements. At the age of 18, she was started on ezetimibe; as her twin sister, after 3 years she switched to combined therapy with the addition of atorvastatin 10 mg/day. No other drugs were used. AUDIT score at the age of 22 was 5, and at the age of 28 was 3. Patients 1 and 2 were both already treated with ezetimibe at the time of enrollment the phase 3 sebelipase alpha trial and they were not enrolled since their alanine aminotransferase(ALT) levels already were less than 1.5 times the upper limit of normal (eligibility criterium for enrollment).

Patient 3 was born from unrelated parents. Family history of dyslipidemia was not reported. He was diagnosed during evaluation for inguinal lymphadenopathy associated with increased hepatic transaminases and mild hepatosplenomegaly, unresponsive to anti-inflammatory and antibiotic treatment. At the time of diagnosis, hepatic elastography by Fibroscan demonstrated mild fibrosis (stiffness kPa 6.8; reference values: stiffness < 5 kPa corresponding to Metavir score F0, stiffness < 8.9 kPa to Metavir score F1). Liver biopsy showed enlargement of hilar periportal spaces secondary to fibrosis, and obstruction by foamy and pigmented histiocytes, with mild lymphoid infiltration, highly suggestive for LAL deficiency. Immediately after the diagnosis, he was treated with ezetimibe at daily-dose of 10 mg/day for 10 years, without addition of statin; no adverse events were recorded. No other drugs were used. He denied alcohol consumption.

## Results

**Fig. 2:**
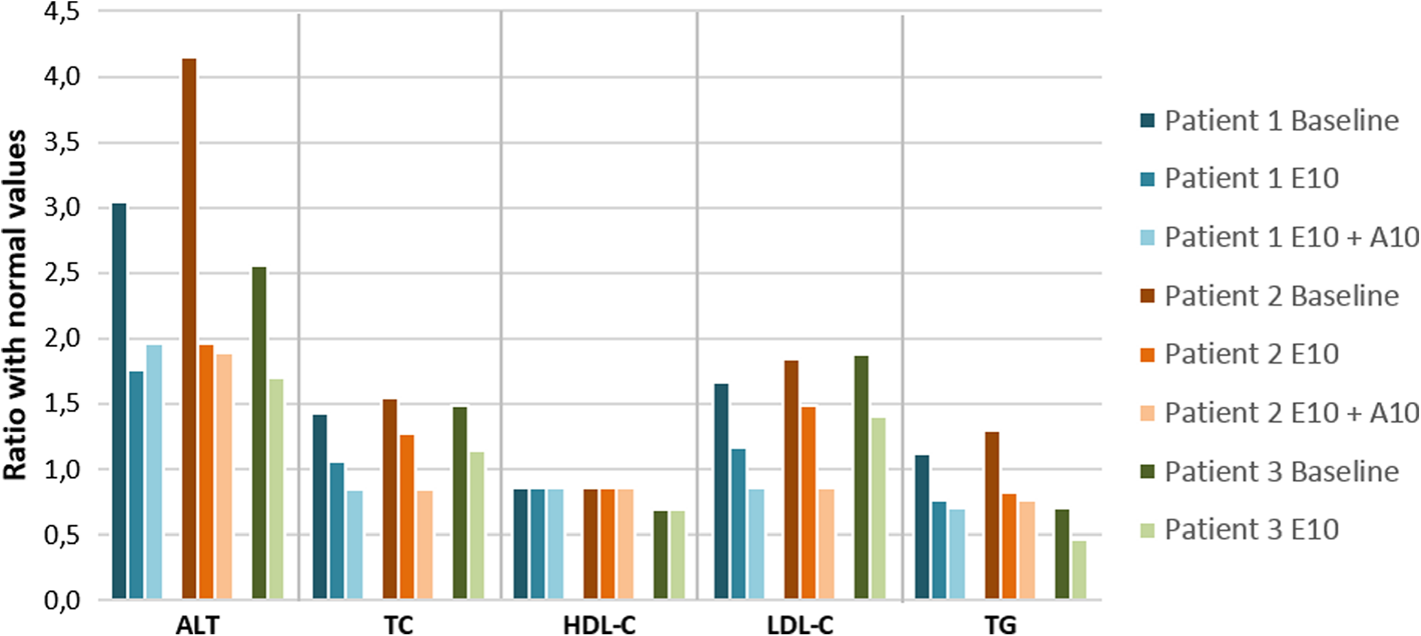
Baseline values (calculated as mean values of the 3 years preceding the start of treatment) in comparison with mean values during ezetimibe treatment and during ezetimibe and statins treatment of Alanine transaminase (ALT), Total Cholesterol (TC), LDL- Cholesterol (LDL-C), HDL Cholesterol (HDL-C), Triglycerides (TG). In order to make data comparable, values are expressed as a ratio with maximum or minimal normal values for age according to our laboratory. ALT reference 10-45 IU/L; Total Cholesterol (TC) reference value below 5.17 mmol/L, Triglycerides (TG) reference value 0.3–1.7 mmol/L, HDL cholesterol (HDL-C) reference value above 1.16 mmmol/L, LDL cholesterol (LDL-C) reference value below 3.36 mmol/L. E10 = ezetimibe 10 mg/day; A10 = atorvastatin 10 mg/day

After 9 years of treatment in Patients 1 and 2, and 10 years of treatment in Patient 3, all three subjects showed reduction of ALT, TC and TG (Table 2, Fig. 2) and mild liver fibrosis by hepatic elastography with Fibroscan: Patient 1 stiffness 7.0 kPa; Patient 2 stiffness 7.9 kPa; Patient 3 stiffness 7.0 kPa.

**Table 2:** Biochemical data before and after ezetimibe treatment

	Patient 1			Patient 2			Patient 3	
	Baseline	E10	E10 + A10	Baseline	E10	E10 + A10	Baseline	E10
**ALT** (IU/L)	137.2 ± 37.1	79.1 ± 12.8**	88.5 ± 28.5**	186.7 ± 41.8	88.2 ± 26.5**	84.9 ± 21.1**	115.0	76.3 ± 7.2
**TC** (mmol/L)	7.36 ± 0.75	5.54 ± 0.50**	4.40 ± 0.82**†	7.98 ± 0.84	6.59 ± 1.22**	4.37 ± 0.42**†	7.70	5.92 ± 0.44
**HDL-C** (mmol/L)	0.96 ± 0.07	1.01 ± 0.06	0.95 ± 0.06	0.95 ± 0.07	0.96 ± 0.05	0.93 ± 0.07	0.82	0.80 ± 0.04
**LDL-C** (mmol/L)	5.60 ± 0.77	3.94 ± 0.55**	2.92 ± 0.78**†	6.17 ± 0.80	5.02 ± 1.19*	2.87 ± 0.40**†	6.34	4.71 ± 0.42
**TG** (mmol/L)	1.94 ± 0.62	1.25 ± 0.24*	1.18 ± 0.43*	2.24 ± 0.47	1.43 ± 0.63*	1.29 ± 0.45**	1.25	0.82 ± 0.19

Baseline values were calculated as mean values of the 3 years preceding the start of treatment for patient 1 and patient 2 and as value at the diagnosis, before the start of treatment for patient 3; E10 = ezetimibe 10 mg/day; A10 = atorvastatin 10 mg/day. Wilcoxon test: *P < 0.05 vs baseline; **P < 0.02 vs baseline; †*P* < 0.02 vs E10. ALT reference 10–45 IU/L; Total Cholesterol (TC) reference value below 5.17 mmol/L, Triglycerides (TG) reference value 0.3–1.7 mmol/L, HDL cholesterol (HDL-C) reference value above 1.16 mmmol/L, LDL cholesterol (LDL-C) reference value below 3.36 mmol/L

## Discussion

Here we report a long term study of 3 patients affected by LAL-D treated with ezetimibe alone or associated with statin.

The target of ezetimibe is NPC1L1 protein, which is widely present in many human tissues, with highest expression in small intestine and in liver. The inhibition of NPC1L1 by ezetimibe reduces the mobilization of CE and TG to the liver through the receptor-mediated uptake of chylomicron remnant particles by decreasing cholesterol absorption in the small intestine and entero-hepatic circulation of cholesterol. It also reduces liver cholesterol stores, and increases LDL clearance with final effect of decreasing plasma LDL-C levels [9].

Being LDLs and the other apoB-containing lipoproteins the substrate of lysosomal acid lipase, the mechanism of action of ezetimibe could be considered similar to that of other drugs acting as substrate reduction therapy. Substrate reduction therapy is based on the concept that, in case of a defect of a catalytic enzyme, the reduction of substrate molecules flux reduces the storage of these molecules. An example of this treatment is eliglustat, licensed for Gaucher disease [10].

In LAL-D, as well as in other lysosomal diseases, enzyme deficiency is not only the cause of storage of under-degraded molecules, but also the reason of generalized lysosome dysfunction, impaired autophagy, and inflammasome activation leading to hepatic fibrosis [11]. Abnormal activation of innate immunity is also reported in non-alcoholic steatohepatitis (NASH) and non-alcoholic fatty liver disease (NAFLD), which require NOD-like receptor protein 3 (NLRP3) inflammasome activation for fibrosis development [12].

In animal model of LAL-D and in a patient with LAL-D, as well as in NAFLD and NASH patients, ezetimibe is reported to be effective to reduce hepatic disease.

In *lal−/−* mice treated with ezetimibe (20 mg/day/kg/bw) for 4 weeks, starting from 21 days of life, Chuang demonstrated a significant decrease of both liver mass and liver cholesterol concentration, as well as ALT levels [13]. Data about plasma cholesterol in *lal−/−* mice were not significant because, differently from humans, the animal model does not develop dyslipidemia. More recently, Chuang measured rates of cholesterol synthesis in vivo in the liver and in small intestine of *lal−/−* mice treated with ezetimibe; he demonstrated a reduction of esterified cholesterol storage in the liver and in the small intestine, even though the rates of liver and small intestine cholesterol synthesis were comparable or exceeded those in matching untreated *lal−/−* mice. These data show the importance of cholesterol intestinal absorption for the storage of cholesteryl esters in the liver of *lal−/−* mice [14]. Furthermore, in animal models ezetimibe ameliorates steatohepatitis by autophagy induction through AMP protein kinase activation, transcriptional EB nuclear factor translocation, and NLRP3 inflammasome inhibition [15]. Therefore, ezetimibe has a double effect on the liver, by reducing CE storage in hepatocyte, and by inhibiting inflammasomes, which lead to liver fibrosis.

There are only two full reports of LAL-D patient treated with ezetimibe. In a 18 year-old CESD subject, treated with ezetimibe associated with lovastatin (40 mg/day) for 12 months, the combined treatment not only resulted in a significant reduction of plasma concentration of TC and TG, but also in reduction of ALT and liver size, suggesting a decrease in the CE accumulation [16].

Cameron reported a 35 year-old woman, followed over two decades and previously treated with atorvastatin or rosuvastatin, and subsequently with the combination of atorvastatin and ezetimibe (40 and 10 mg/day, respectively) for 2 years, with normalization of lipid profile and liver function tests [17].

Besides, the efficacy of ezetimibe on hepatic disease has been shown in NAFLD, NASH and related conditions in humans [18–24].

Concerning the effect of ezetimibe in lipid lowering, even if ezetimibe is indicated for primary hypercholesterolemia, it is often used as an add-on therapy, being statins more potent lipid lowering medications and first line treatment of dyslipidemias. Bernestein reviewed 132 CESD patients, 35 of them were treated with statins for hyperlipidemia. Fifteen patients treated with statins had biopsy findings with fibrosis, cirrhosis or other typical pathologic abnormalities CESD-associated, but no long-term or sequential follow-up data reported. The other 12 patients had multiple liver biopsies, but there were no cases whose liver histology improved; 6 patients required transplantation or died from liver failure. These findings emphasize the lack of efficacy of statins in ameliorating liver disease or preventing its progression even if TC and LDL-C decrease [25].

The patients here reported have been treated with ezetimibe for 9 and 10 years, with a significant reduction of ALT level as well as a reduction of total cholesterol, LDL-C and TG levels. None of the patients was followed by liver biopsy and we cannot exclude spontaneous improvement in ALT as liver cirrhosis is found in histology, but no progression of liver disease is demonstrated in our three patients, who only have a mild fibrosis after 9 years (patient 1 and patient 2) and 10 years (patient 3) of treatment on Fibroscan elastography.

Concerning the control of hyperlipidemia, ezetimibe was associated with statin in the patient 1 and in the patient 2, but retrospectively we are unsure about the need to add statin. The clinical outcome observed in patient 3,who was treated with ezetimibe alone, raises the question if there was further benefit in adding a statin.

The ALT levels reduction was the primary end point of clinical trial with sebelipase alpha, whereas the reduction of TC, LDL-C, and TG levels were secondary end points of the same study [8]. Therefore, it is appropriate to compare the efficacy of ezetimibe with ERT efficacy.

Clinical trials have demonstrated that sebelipase alpha is efficacy in reducing these surrogate outcomes but it is uncertain from the original clinical trial data whether sebelipase alpha delays or stops disease progression to cirrhosis, hepatocellular carcinoma, and need for liver transplant. Another important issue is related to managing access to ERT.

In infants under 6 months, LAL-D is rapidly progressive and associated with a very short life expectancy. On the other hand, disease progression is highly variable in patients presenting with symptoms of LAL-D in childhood or adulthood. Therefore, there is wide consensus in ERT for infants because it is the only treatment that can prevent early death, but there is an open discussion about treatment in patients whose symptoms are less severe and whose condition is more slowly progressive.

Finally, the high cost of treatment with sebelipase alpha poses a significant obstacle for some national health system plans.

Based on the above, ezetimibe could be an alternative oral therapy to ERT, while the association ERT-ezetimibe or statins proposed by Block [26] seems not appropriate mostly because of the cost.

## Conclusion

In this observational case series ezetimibe is an effective, safe, and sustainable treatment for LAL-D. Further studies are warranted to demonstrate that ezetimibe is an alternative therapy to enzyme replacement therapy.

## Abbreviations

ABCA1: Adenosine Triphosphate-Binding Cassette Transporter A1
ALT: Alanine Amino Transferase
AUDIT: Alcohol Use Disorders Identification Test
CE: Cholesteryl Esters
CESD: Cholesteryl Ester Storage Disease
ERT: Enzyme Replacement Therapy
HDL-C: High Density Lipoprotein Cholesterol
LAL-D: Lysosomal Acid Lipase Deficiency
LDL-C: Low Density Lipoprotein Cholesterol
NAFLD: Non-Alcoholic Fatty Liver Disease
NASH: Non-Alcoholic Steato Hepatitis
NLRP3: NOD-like receptor protein 3
NPC1L1: Niemann Pick C1-like 1
TC: Total Cholesterol
TG: Triglycerides
WD: Wolman Disease

## References

[1] Hoffman EP, Barr ML, Giovanni MA, Murray MF Lysosomal Acid Lipase Deficiency. In: In: Pagon RA, Adam MP, Ardinger HH, Wallace SE, Amemiya A, Bean LJH, Bird TD, Ledbetter N, Mefford HC, Smith RJH, Stephens K, editors. GeneReviews® [Internet]. Seattle (WA): University of Washington, Seattle; 1993–2017. 2015 Jul 30 [updated 2016 Sep 1].

[2] Jones SA, Valayannopoulos V, Schneider EES, Banikazemi M, Bialer M, Cederbaum S, Chan A, Dhawan A, Di Rocco M, Domm J, Enns GM, Finegold D, Gargus JJ, Guardamagna O, Hendriksz C, Mahmoud IG, Raiman J, Selim LA, Whitley CB, Zaki O, Quinn AG (2016). Rapid progression and mortality of lysosomal acid lipase deficiency presenting in infants. Genet Med.

[3] Burton BK, Deegan PB, Enns Barić I, Burrow TA, Camarena Grande C, Coker M, Consuelo-Sánchez A, Deegan P, Di Rocco M, Enns GM, Erbe R, Ezgu F, Ficicioglu C, Furuya KN, Kane J, Laukaitis C, Mengel E, Neilan EG, Nightingale S, Peters H, Scarpa M, Schwab KO, Smolka V, Valayannopoulos V, Wood M, Goodman Z, Yang Y, Eckert S, Rojas-Caro S, Quinn AG (2015). Clinical features of Lysosomal Acid Lipase Deficiency - a longitudinal assessment of 48 children and adults. J Pediatr Gastroenterol Nutr.

[4] Pisciotta L, Fresa R, Bellocchio A, Pino E, Guido V, Cantafora A, Di Rocco M, Calandra S, Bertolini S (2009). Cholesteryl Ester storage disease (CESD) due to novel mutations in the LIPA gene. Mol Genet Metab.

[5] Reiner Ž, Guardamagna O, Nair D, Soran H, Hovingh K, Bertolini S, Jones S, Ćorić M, Calandra S, Hamilton J, Eagleton T, Ros E (2014). Lysosomal acid lipase deficiency--an under-recognized cause of dyslipidaemia and liver dysfunction. Atherosclerosis.

[6] Zhang B, Porto AF (2013). Cholesteryl ester storage disease: protean presentations of lysosomal acid lipase deficiency. J Pediatric Gastroenterol Nutr.

[7] Zimetti F, Favari E, Cagliero P, Adorni MP, Ronda N, Bonardi R, Gomaraschi M, Calabresi L, Bernini F, Guardamagna O (2015). Cholesterol trafficking-related serum lipoprotein functions in children with cholesteryl ester storage disease. Atherosclerosis.

[8] Burton BK, Balwani M, Feillet F, Barić I, Burrow TA, Camarena Grande C, Coker M, Consuelo-Sánchez A, Deegan P, Di Rocco M, Enns GM, Erbe R, Ezgu F, Ficicioglu C, Furuya KN, Kane J, Laukaitis C, Mengel E, Neilan EG, Nightingale S, Peters H, Scarpa M, Schwab KO, Smolka V, Valayannopoulos V, Wood M, Goodman Z, Yang Y, Eckert S, Rojas-Caro S, Quinn AG (2015). A phase 3 trial of Sebelipase Alfa in Lysosomal acid lipase deficiency. N Engl J Med.

[9] Kosoglou T, Statkevich P, Johnson-Levonas AO, Paolini JF, Bergman AJ, Alton KB (2005). Ezetimibe: a review of its metabolism, pharmacokinetics and drug interactions. Clin Pharmacokinet.

[10] Cox TM, Drelichman G, Cravo R, Balwani M, Burrow TA, Martins AM, Lukina E, Rosenbloom B, Goker-Alpan O, Watman N, El-Beshlawy A, Kishnani PS, Pedroso ML, Gaemers SJM, Tayag R, Peterschmitt MJ (2015). Eliglustat compared with imiglucerase in patients with Gaucher’s disease type 1 stabilised on enzyme replacement therapy: a phase 3, randomised, open-label, non-inferiority trial. Lancet.

[11] Rigante D, Cipolla C, Umberto Basile U, Gulli M, Savastano MC (2017). Overview of immune abnormalities in lysosomal storage disorders. Immunol Lett.

[12] Wree A, MD MG, Peña CA, Schlattjan M, Li H, Inzaugarat ME, Messer K, Canbay A, Hoffman HM, Feldstein AE (2014). NLRP3 inflammasome activation is required for fibrosis development in nafld. J Mol Med.

[13] Chuang JC, Lopez AM, Posey KS, Turley SD (2014). Ezetimibe markedly attenuates hepatic cholesterol accumulation and improves liver function in the lysosomal acid lipase-deficient mouse, a model for cholesteryl ester storage disease. Biochem Biophys Res Commun.

[14] Chuang JC, Lopez AM, Turley SD (2017). Quantitation of the rates of hepatic and intestinal cholesterol synthesis in lysosomal acid lipase-deficient mice before and during treatment with ezetimibe. Biochem Pharmacol.

[15] Kim SH, Kim G, Han DH, Lee M, Kim I, Kim B (2017). Ezetimibe ameliorates steatohepatitis via AMP activated protein kinase-TFEB-mediated activation of autophagy and NLRP3 inflammasome inhibition. Autophagy.

[16] Tadiboyina VT, Liu DM, Miskie BA, Wang J, Hegele RA (2005). Treatment of dyslipidemia with lovastatin and ezetimibe in an adolescent with cholesterol ester storage disease. Lipids Health Dis.

[17] Cameron SJ, Daimee U (2015). Block RC a case of abdominal pain with dyslipidemia: difficulties diagnosing cholesterol ester storage disease. Eur Rev Med Pharmacol Sci.

[18] Yoneda M, Fujita K, Nozaki YEndo H, Takahashi H, Hosono K, Suzuki K, Mawatari H, Kirikoshi H, Inamori M, Saito S, Iwasaki T, Terauchi Y, Kubota K, Maeyama S, Nakajima A (2010). Efficacy of ezetimibe for the treatment of non-alcoholic steatohepatitis: an open-label, pilot study. Hepatol Res.

[19] Chan DC, Watts GF, Gan Y, Ooi EM, Barrett PH (2010). Effect of ezetimibe on hepatic fat, inflammatory markers, and apolipoprotein B-100 kinetics in insulin-resistant obese subjects on a weight loss diet. Diabetes Care.

[20] Takeshita Y, Takamura T, Honda M, Kita Y, Zen Y, Kato K, Misu H, Ota T, Nakamura M, Yamada K, Sunagozaka H, Arai K, Yamashita T, Mizukoshi E, Kaneko S (2014). The effects of ezetimibe on non-alcoholic fatty liver disease and glucose metabolism: a randomised controlled trial. Diabetologia.

[21] Park H, Shima T, Yamaguchi K, Mitsuyoshi H, Minami M, Yasui K, Itoh Y, Yoshikawa T, Fukui M, Hasegawa G, Nakamura N, Ohta M, Obayashi H, Okanoue T (2011). Efficacy of long-term ezetimibe therapy in patients with nonalcoholic fatty liver disease. J Gastroenterol.

[22] Enjoji M, Machida K, Kohjima M, Kato M, Kotoh K, Matsunaga K, Nakashima M, Nakamuta M (2010). NPC1L1 inhibitor ezetimibe is a reliable therapeutic agent for non-obese patients with nonalcoholic fatty liver disease. Lipids Health Dis.

[23] Shiwa T, Kawanami Y, Yokoyama T, Moritani A, Hashimoto M, Gotoh T (2011). The efficacy of ezetimibe on nonalcoholic fatty liver disease (NAFLD). Nihon Shokakibyo Gakkai Zasshi.

[24] Nakade Y, Murotani K, Inoue T, Kobayashi Y, Yamamoto T, Ishii N, Ohashi T, Ito K, Fukuzawa Y, Yoneda M. Ezetimibe for the treatment of non-alcoholic fatty liver disease: a meta-analysis. Hepatol Res. 2017; 10.1111/hepr.12887. 10.1111/hepr.12887 28257594

[25] Bernstein DL, Hulkova H, Bialer MG, Desnick RJ (2013). Cholesteryl ester storage disease: review of the findings in 135 reported patients with underdiagnosed disease. J Hepatol.

[26] Block RC, Razani B (2016). Options to consider when treating lysosomal acid lipase deficiency. J Clin Lipidol.

